# Foraging Habitat Availability and the Non-Fish Diet Composition of the Grey Heron (*Ardea cinerea*) at Two Spatial Scales

**DOI:** 10.3390/ani14172461

**Published:** 2024-08-24

**Authors:** Karolina Cieślińska, Brygida Manikowska-Ślepowrońska, Adam Zbyryt, Dariusz Jakubas

**Affiliations:** 1 Department of Vertebrate Ecology and Zoology, Faculty of Biology, University of Gdańsk, ul. Wita Stwosza 59, 80-308 Gdańsk, Poland; karolina.cieslinska7@gmail.com (K.C.); biodj@univ.gda.pl (D.J.); 2 Doctoral School of Exact and Natural Sciences, University of Białystok, ul. Konstantego Ciołkowskiego 1K, 15-245 Białystok, Poland; adam.zbyryt@gmail.com

**Keywords:** colonial nesting, indicator species, land cover, opportunistic feeding, pellets

## Abstract

**Simple Summary:**

In this study, we investigated differences in the non-fish diet components of the Grey Heron (*Ardea cinerea*) at both regional and local scales. We found significant inter-regional and inter-colony differences, not only in the diet but also in the habitat composition of foraging areas. We also identified significant relationships between some prey items and different habitats. This study shows that the opportunistic character of the diet of the Grey Heron is adapted to the local foraging habitat and the availability of non-fish prey.

**Abstract:**

Habitat structure on foraging ground is one of the crucial factors determining diet diversity in colonially breeding avian predators. Quantifying the habitat and diet composition at different spatial scales (regional and local inter-colonial) can help provide understanding of the drivers of diet composition. In this study, we examined the composition of the non-fish diet of an opportunistic predator, the Grey Heron (*Ardea cinerea*), based on pellets. We compared pellets from 21 colonies in two different regions of Poland that differing in composition of foraging habitats. Multivariate statistical techniques were used to analyze the relationship between diet and habitat compositions in a 20 km radius around these colonies. Significant inter-regional and inter-colony differences in Grey Heron diet and habitat composition were detected in foraging areas. However, some prey were present in the diet only from one of the regions. Around the heronries with a predominance of farmland habitats, the European water vole (*Arvicola amphibius*) and terrestrial invertebrates were present in relatively low frequencies, and the striped field mouse (*Apodemus agrarius*) was present in high frequencies. Voles (*Microtus* sp.) were more frequent in colonies with prevalence of non-irrigated arable land around the colony, in contrast to the bank vole (*Myodes glareolus*), which was less common in heronries with a higher contribution of this habitat type. Remains of aquatic invertebrates were less abundant in colonies surrounded by extensive forests. The results of our research indicate the opportunistic character of the non-fish part of the diet of the Grey Heron adapted to the local foraging habitat and prey availability.

## 1. Introduction

Knowledge about species–habitat relations is important for understanding processes occurring within natural ecosystems. Habitat has a significant impact on the development and survival of those inhabiting it [1]. The availability of aquatic habitats is crucial for the occurrence of waterbirds. The Grey Heron (*Ardea cinerea*) is an example of a colonially breeding waterbird that explores various habitats, including aquatic ecosystems [2,3,4]. Its diet consists mainly of fish and is supplemented by many other aquatic, semi-aquatic, and terrestrial species, i.e., invertebrates, amphibians, reptiles, mammals, and birds [2,3,5,6]. Their diet composition is variable and is mainly dependent on local prey availability [5,6], individual prey preferences, and/or time of the year [2,7,8,9]. Studies on the Grey Heron’s diet composition are usually based on regurgitated food [2,3,5,6] or the analysis of pellets [undigested food items comprising, i.e., mammalian hairs, feathers, skeletal elements, and invertebrate remains (mainly elytra)]. Due to a mainly piscivorous diet [2], Grey Herons have evolved an extremely efficient digestive process towards fish, which results in a lack of this type of prey remains in pellets (e.g., [10]). In contrast, integument remains of secondary prey types, e.g., small mammals (fur) and invertebrates (exoskeleton) [2], are hard to break down due to digestive-resistant chemical components, i.e., keratin and chitin, respectively [11], resulting in their frequent presence in pellets [3]. Even though these supplementary diet components make up a smaller proportion of the total biomass consumed, they may still serve as a great important source of some nutrients and microelements that are absent in the main part of the diet [8].

Colonies of Grey Herons are not randomly located [4]. According to optimal foraging theory [12,13], an optimal nesting site must fulfill the requirements to successfully accomplish breeding, including the provision of access to prey-abundant foraging areas [4]. Also, foraging areas should be located at a cost-effective distance from the colony, securing energetical profits for individuals both for commuting as well as foraging [13,14]. Because of their structure, vegetation composition and distribution, or prey species associated with them, various foraging habitat types are characterized by differences in prey availability [15].

In this study, we examined differences in the frequency of occurrence of prey items in Grey Heron pellets at two spatial scales: (1) inter-regional differences reflected in the presence of some unique foraging habitats in one of the two regions we studied; and (2) local differences comprising different habitat composition in each colony’s actual foraging areas (independently of regions).

We expect that the frequency of occurrence of different prey items in pellets will differ at both spatial scales. Firstly, the presence of unique habitat types in one of the regions should be reflected in differences in diet composition. In Dutch colonies of Grey Herons, it was expressed by the less frequent presence of aquatic invertebrates in pellets collected in the coastal colonies [5]. Secondly, the composition of common habitats around particular colonies may affect prey composition. For example, aquatic invertebrates are more commonly reported from colonies with a higher abundance of hydrographic networks or wetlands [16].

## 2. Materials and Methods

### 2.1. Study Area Regions

**Figure 1:**
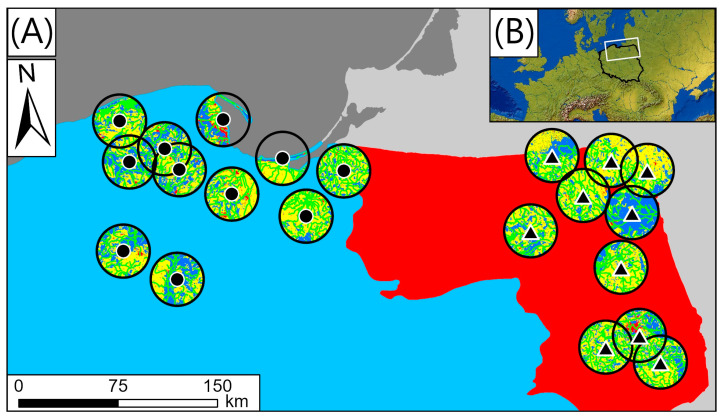
Distribution of heronries in northern (black circles) and northeastern Poland (black triangles) (**A**) and location of the studied area in Europe (**B**). Each circle represents a 20 km buffer zone from the center of each colony. Colors within buffer zones stand for various habitat types, i.e., blue—forest (potential nesting sites), green—foraging areas optimal for Grey Herons (hydrographic features, wetlands, coastal zone), yellow—suboptimal foraging areas (farmlands, grassy, open areas, etc.), red—urban zones (avoided by Grey Heron). Two color zones symbolize Poland megaregions adapted from [18]: Beyond-Alps Europe (blue) and East European Plain (red). Map source: naturalearthdata.com (accessed on 30 September 2023).

Both regions are located in the temperate zone in Central Europe in Poland. The northern (N) region constitutes a part of the Beyond-Alps Europe [17,18] and includes the coast of the Baltic Sea with the Hel Peninsula and Vistula Split, the Gdańsk Bay, and the Vistula Lagoon. The landscape of the coastal zone comprises river mouths and salt marshes and is specific to the N region. The Eastern part of this region comprises Żuławy Alluvial Plain with a deforested agricultural landscape, in large part reclaimed artificially by dykes, pumps, channels, and extensive drainage systems [19,20]. The inland area with lake districts is characterized by a post-glacial landscape with moraine hills (up to 309 m) and postglacial lakes, as well as depressions, basins, and valleys [21]. Seven heronries are inland and four are coastal (Będziechowo, Kąty Rybackie, Kąty Rybackie, Płoskinia; see a full list of analyzed colonies, name abbreviations, and their localization in the Supplementary Material—S1), i.e., contain a SEACO habitat within 20 km of colonies, equivalent to being within the range of foraging flights [10,22] (Figure 1).

The NE region constitutes a part of the East European Plain. The landscape is characterized by the presence of numerous narrow, deep, ribbon-shaped lakes and wide melt-holes embedded in medial moraine depressions. Lakelands, lowlands including lakeless old glacial high plains, young glacial plains (mostly containing lakes), and outwash plains with lakes are also present. The greater part of this area is covered by extensive plains on the site of former stagnant reservoirs [21]. The lakeless Podlasko-Belarussian belt of uplands is characterized by extensive marshy depressions [21]. This part of the megaregion (East European Plain) is also characterized by abundant areas of mixed forests constituting a part of the Palearctic terrestrial ecoregion (Central European mixed forests) [23] (Figure 1).

### 2.2. Data Collection and Analyses

We collected pellets in the field during the 2016 breeding season in heronries of the Grey Heron in northern (*n =* 11) and northeastern (*n =* 10) Poland, representing two different physico-geographical megaregions [18] (Figure 1). We collected pellet material from under nest trees two to four times during the middle and late phases of the chick-rearing period, between the second week of May and the end of June. Ten colonies in the north comprise only herons; only the colony at Kąty Rybackie was mixed with Great Cormorants (*Phalacrocorax carbo*). Nine of the northeastern colonies comprised only herons; only the colony at Gołdap was mixed with Great Cormorants and Great Egrets (*Ardea alba*) (see Supplementary Material S1). We did not investigate the influence of either population size and/or intra- and inter-specific competition on the Grey Heron’s diet composition.

In total, we collected and analyzed 210 pellets from 21 colonies (see the full description of the heronries, including colony name, abbreviation, longitude, latitude, and name origin, in Supplementary Data S1). Immediately after these colony visits, we froze pellets at −20 °C to prevent the degradation of biological material. Then, after defrosting the pellets, we removed all bones, invertebrate remains, feathers, and other solid parts for species identification. We identified mammal skulls following the keys to vertebrates from [24]. We randomly sampled ten hairs for microscopic analysis. For prey species identification, we considered cuticula’s scale and medullary and sectioned hair patterns using the key of [25]. We prepared hair samples for identification by the methods proposed by [26]. We identified invertebrate remains using the following literature: [27,28]. To ensure consistency, one person identified all vertebrate (B.M.-Ś.) and invertebrate (Mateusz Krawczuk) elements of pellets. Due to the preserved condition of these extracted prey remains, we identified them with varying levels of taxonomic detail (from phylum to species). As herons possess an extremely efficient digestive system [29], the majority of fish bones, scales, and otoliths are digested completely [5]. Thus, we did not find these types of remains in the collected material.

### 2.3. Pellet Analyses

Presence status for each taxon was established by a minimum single observation of its remains within a single pellet, i.e., present = 1; absent = 0. The frequency of taxon occurrence was calculated based on the presence/absence ratios among all collected pellets at a colony (*n =* 11 in N Poland and *n =* 10 in NE Poland) and region (N Poland: *n*/110; NE Poland: *n*/100).

In total, we distinguished 21 non-fish prey item categories (Table 1), and we excluded prey items with low frequency (*n* < 2) (except for invertebrates; see below). We grouped invertebrate remains into two categories based on the environment mostly inhabited during the life cycle (aquatic/terrestrial) (Table 1). Such an approach enabled us to successfully distinguish groups of invertebrates preyed on by Grey Herons in particular habitat types.

We divided invertebrate taxa as follows:

(A) Fully/mostly aquatic: *Acilius* sp., Bivalvia, *Carcinus maenas*, *Colymbetes fuscus*, Corixidae, *Dytiscus marginalis*, Dytiscidae, Dytiscidae larvae, Haliplidae, *Haliplus confinis*, *Ilyocoris cimicoides*, Hydrophilidae, Naucoridae, Nepomorpha, Notonectidae, and Odonata;
(B) Terrestrial: Carabidae, Curculionidae, *Georissus* sp., *Graphosoma* sp., *Gryllotalpa gryllotalpa*, *Melolontha melolontha*, *Omophron limbatum*, and Scarabeidae.

This applied approach enabled us to distinguish groups of invertebrates related to optimal foraging grounds, e.g., hydrographic networks, wetlands, and suboptimal foraging areas, e.g., open grassy spaces or agro-related habitats [4].

### 2.4. Habitat Composition Analyses

We derived information about 10 types of habitats (environmental variables) in the vicinity of heronries from Corine Land Cover 2018 [30] (hereafter CLC) [31]. Inland hydrographic network features (waterbodies, rivers) were derived from the European Environment Agency dataset [32]. We used this dataset since it characterizes a higher level of detail than CLC, which is important during the evaluation and analysis of optimal foraging grounds. We used data about the area of the following habitats from land cover level 3 of CORINE [4]:

(1) Hydrographic network: river/canals, water bodies (i.e., lakes, ponds), inland wetlands (e.g., peat bogs), and an area of the coastal zone (2 m wide; present within the foraging range of four studied colonies). Hydrographic networks serve as important foraging areas for Grey Herons during the breeding season [33], thus constituting an important landscape factor affecting the location of breeding colonies of this species [4,14,34];
(2) Complex cultivation patterns, land principally occupied by agriculture, with significant areas of natural vegetation, non-irrigated arable land, pastures which are area of suboptimal foraging grounds;
(3) Forests (all types combined), often serving as nesting habitats;
(4) Urban zones (airports, construction sites, continuous urban fabric, discontinuous urban fabric, dumpsites, industrial or commercial units, mineral extract sites, port areas, road and rail networks, and sport and leisure facilities), which may be avoided by herons.

In spatial analyses, we calculated the area of each habitat in a 20 km radius around colonies (Table 2), following [22]. Such a distance corresponds to the maximal range of foraging trips by Grey Herons (e.g., [4,22,34,35,36]).

Due to the lack of coverage of CORINE for the Kaliningrad Oblast (Russia) bordering northeast Poland, we were not able to extract full habitat type cover for two colonies (Płoskinia in N Poland and Gołdap in NE Poland). To obtain land cover data for the Kaliningrad Oblast, we used deep learning-based, semantic classification based on already known CLC classification overlapping Sentinel 2 imagery with the help of ArcGIS Pro image analyst tools [37]. The deep learning algorithm was based on manually selected training samples (*n =* 621) based on clear satellite imagery from S2MSIC products the peak of vegetation season (August) [38]. In order to remove the negative effects of the atmosphere on satellite imagery quality (scattering or absorption of sensed light) [39,40,41], we conducted DOS 1 atmospheric correction (Dark Object Subtraction 1) for each band individually [40]. We used three bands from Sentinel 2 imagery (band number; ‘name’; [central wavelength]): (B2; ‘blue’; [490 nm]; B3; ‘green’; [560 nm]; B4; ‘red’; [665 nm]), to create natural color multiband raster (bands were set in the following order: B4; B3; B2) [42]. Combinations of these channels were most efficient for recognizing particular types of land cover, e.g., mature crop fields, forests, bare ground, urban areas, and water bodies, thus providing us with the most satisfying results.

DOS 1 atmospheric correction was conducted via the Semi-Automatic Classification Plugin for QGIS (developed by Luca Congedo) [41] in QGIS 3.18 (QGIS Geographic Information System. QGIS Association). Manual classification was performed with ArcGIS Pro 3.0.3 Classification Wizard tools (Esri, Redlands, CA, USA) [37]. All spatial GIS analyses were performed in ArcGIS Pro 3.0.3 (Esri, Redlands, CA, USA) [37].

### 2.5. Statistical Analyses and Data Preparation

To compare frequencies of particular prey items in pellets and particular habitat areas (km^2^), we performed a series of G-tests of independence (we used frequency of occurrence, i.e., number of cases and their potential vs. maximal frequency of occurrence, i.e., *n =* 110 for the N region and *n =* 100 for the NE region). For cases with low occurrence rate, i.e., n < 5 in each region—we performed the Fisher’s Exact Test, and we reported only *p* value in results using R software ver. 4.2.2 [43].

We did not perform statistical analyses for habitats or prey items present in only one region.

To investigate differences in diet composition between coastal (*n =* 4) and inland (*n =* 7) heronries in the N region, we conducted a series of G tests of independence (we used observed frequencies of occurrence vs. their potential, and maximal frequency of occurrence, i.e., *n =* 40 for coastal zone and *n =* 70 for inland zone. We excluded taxa with occurrence rate n < 5 in both zones to avoid calculation biases. We also did not include taxa that were absent in one of the zones. Nine prey items (50% of all prey taxa from this region) fulfilled our minimum criteria. All calculations were conducted using the *RVAideMemoire* package ver. 0.9−83−7 [44] in R software ver. 4.2.2 [43].

### 2.6. Prey–Habitat Relationship

To test the relationship between habitats and prey items, we performed various types of statistical analyses depending on the quality of prey data (number of documented presences of prey items from every colony in both regions). We used Generalized Linear Models (GLMs) with Poisson distribution with prey frequency in a particular colony as the response variable and the area of particular habitats as explanatory variables. For the cases in which we noted over- or under-fitting with zero values and/or overdispersion, we used GLM with negative binomial regression, as it was the best-fitting model compared to other models, i.e., zero-inflated Poisson regression or zero-inflated negative binomial regression. We checked each model for zero fitting and overdispersion in the performance package [45]. We used the number of pellets per colony as the offset. Firstly, we constructed the full model with all habitat types:

PREY~COMCP+FORES+LANDP+NONIR+PASTU+RIVER+SEACO+WATBO+WETLA

Then, we selected the most parsimonious model using the AIC criterion [46] and the dredge function from the MuMIn package [47]. Due to the relatively small sample size, we restricted the number of predictors in the selected models to two. We only considered models with Δ < 1. We selected the best-fitting model by comparing their AICc values [46]. Negative binomial regression models were created in package MASS [48] in R software ver. 4.2.2. To check the significance of the highest-ranked models, we conducted the Wald test in the *aod* package [49] in R software ver. 4.2.2.

For the striped field mouse recorded exclusively in one region (NE), we performed GLM with binomial distribution to investigate factors affecting the presence/absence of this species in particular colonies. We assessed the performance of the binomial classification model by the Wald test in the *aod* package [49] in R software ver. 4.2.2.

While constructing prey–habitat relationship models, we considered only prey items with n ≥ 40 frequency abundance (at least 36.4% of occurrence probability in both regions), constituting a total of seven cases. To avoid biased results, we did not include smaller data samples.

### 2.7. Multivariate Analyses

To compare the relative area of particular habitats around particular heronries, we used the following multivariate analyses:

(1) Analysis of similarities (ANOSIM) to examine the significance of inter-regional spatial differences [50] in prey item compositions. This analysis is popularly used for testing ecological hypotheses about spatial differences, especially for detecting any environmental impacts on an organism’s assemblages [50]. We applied this method to test for any dissimilarities between regions in prey item contribution and test the impact of environmental variables. ANOSIM creates a value of R between −1 and +1, where 0 represents no impact. Higher positive R values stand for increasing differences between samples [50].
(2) Non–metric multidimensional scaling (nMDS) to summarize patterns of food composition of the diet of the Grey Heron among colonies based on an ordination of pairwise site dissimilarities [51,52] using the Euclidean distance as a measure of dissimilarity and convex hulls. nMDS is an ordination technique using an algorithm that creates the best combination of variables explaining observed differences or similarities among groups [53]. After the ordination procedure conducted by nMDS, prey items and environmental variables were represented on a biplot based on the correlation coefficients between their values and the coordinates of the virtual assemblages of the nMDS axes [54]. The importance of differences among variables was based on calculated coefficients of determination (R^2^) for each of two axes. We conducted this analysis since the stress value was acceptable (<15) [55].
(3) Similarity Percentage (SIMPER) analysis was used to recognize prey items contributing the most to inter-regional differences. In this technique, the general significance of the difference is commonly assessed by ANOSIM. This difference is based on the observed dissimilarity measured in Euclidean distance. For this analysis, we considered cases with a contribution of 10% ≤ of observed differences [55,56].

All multivariate analyses were conducted using PAST software version 4.11 [56].

## 3. Results

### 3.1. Inter-Regional Differences in Diet Composition

In total, 18 prey item types were found in pellets from N Poland and 13 from NE Poland, with 11 prey items exclusively from only one region, i.e., eight in N Poland (*Neomys* sp., Eurasian water shrew, *Crocidura* sp., European edible dormouse, wood mouse, European pine vole, *Sorex* sp., and bird taxa) and three of them in the NE region (*Apodemus agrarius*, *Apodemus* sp., and *Muridae*). We found significant differences in the frequency of six types of prey items between the two regions (Table 3). European water vole, species of genus Microtus, and terrestrial invertebrates were significantly more commonly found in pellets from the N region, while aquatic invertebrates, tundra vole, and yellow-necked mouse were found in the NE region (Table 3). Three taxa were prevalent (i.e., found in above 50% of pellets) in each region. In the N region, European water vole and *Microtus* sp. were prevalent (Table 3), and European mole was prevalent in both regions (with an average frequency of occurrence of 79 in the N region and 67 in the NE region).

### 3.2. Difference in Diet Composition between Coastal and Inland Colonies in the N Region

We did not find any significant differences between coastal and inland heronries in prey item composition. Bird remains were found exclusively in coastal heronries, which also lacked remains of the Eurasian water shrew.

### 3.3. Inter-Regional Differences in Habitats

We found statistically significant differences between regions in proportions of seven of nine habitats found in foraging areas of Grey Herons. First of all, the seacoast was present exclusively in the N region. The N region was also characterized by a higher proportion of forests and non-irrigated arable lands compared to the NE region. The latter region was characterized by a higher proportion of complex cultivation patterns, land principally used for agriculture, pastures, urban habitats, and inland wetlands compared to the N region. The area covered by inland hydrographic networks, i.e., RIVER and WATBO, were similar in both regions (Table 4). NONIR covered the greatest amount of foraging areas of Grey Herons in both regions (above 35% in each region) (Table 4).

### 3.4. Prey–Habitat Relationship

We found significant relationships between the area of particular habitats and the frequency of prey items found in pellets for six out of seven considered prey taxa. The frequency of the European mole (TEU) (*n =* 154) in pellets was not significantly related to the proportion or area of habitat types around heronries (*p* = 0.232).

**Figure 2:**
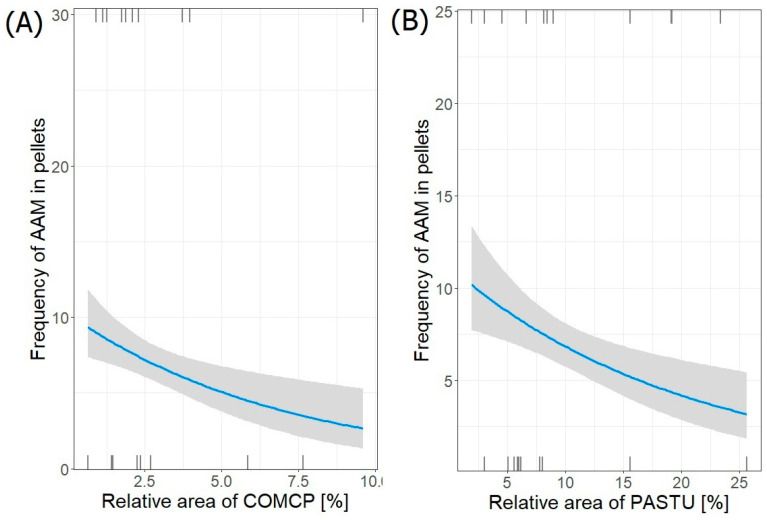
Frequency of water vole (AAM) in pellets from both regions in relation to relative area of (**A**) COMCP and (**B**) PASTU in foraging areas around heronries. Blue lines indicate GLM fit; rug plots of negative (bottom) and positive (top) residuals.

Two best-fit models (GLM with Poisson distribution) describing the relationship between the frequency of occurrence of the water vole (AAM) remains in pellets and its habitats surrounding heronries were selected (Table 5). Its frequency in collected pellets from all colonies (*n =* 140) significantly decreased with both increasing relative area of complex cultivation patterns (COMCP) (estimate ± SE: −0.13993 ± 0.04754; *p* = 0.003) and relative area of pastures (PASTU) around the heronries (−0.04906 ± 0.01565; *p* = 0.002) (Figure 2). In the second-ranked AAM, the frequency increased significantly with relative area of complex cultivation patterns (COMCP) and relative area of pastures (PASTU). It also increased with the presence of (SEACO), but this relationship was not significant (*p* = 0.13). Both selected models were significant (Wald test, *p* < 0.05).

**Figure 3:**
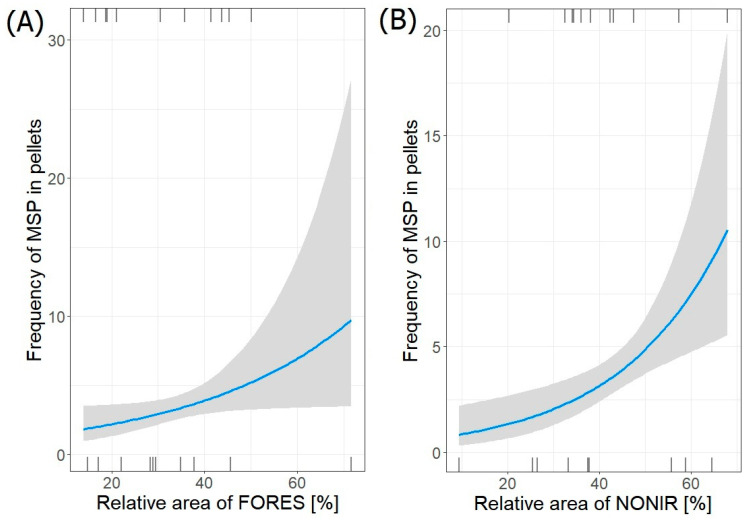
Frequency of Microtus sp. (MSP) in pellets from both regions in relation to relative area of (**A**) FORES and (**B**) NONIR in foraging areas around heronries. Blue lines indicate GLM fit; rug plots of negative (bottom) and positive (top) residuals.

Only one model (GLM negative binominal regression) described the best relationship between the frequency of occurrence of vole sp. (MSP) remains in pellets and habitats surrounding heronries (Table 6). Its frequency in pellets from all colonies (*n =* 77) significantly increased with both increasing relative area of forests (FORES) (estimate ± SE: 0.02893 ± 0.01410; *p* = 0.04) and non-irrigated arable land (NONIR) (0.04323 ± 0.01339; *p* = 0.001). The intercept value was also statistically significant (*p* = 0.0001) (Figure 3). The model was significant (*p* < 0.0001).

**Figure 4:**
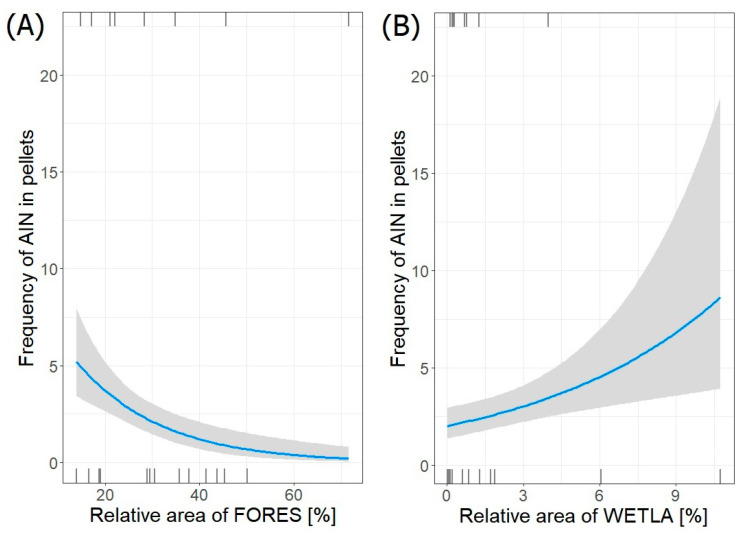
Frequency of aquatic invertebrates (AIN) in pellets from both regions in relation to relative area of (**A**) FORES and (**B**) WETLA in foraging areas around heronries. Blue lines indicate GLM fit; rug plots of negative (bottom) and positive (top) residuals.

Only one best-fit model (GLM negative binominal regression) describing the relation between the frequency of occurrence of aquatic invertebrates (AIN) remains in pellets and its habitats surrounding heronries was selected (Table 7). The frequency of aquatic invertebrates in pellets collected in all colonies (*n =* 63) significantly decreased with increasing relative area of forests (FORES) (estimate ± SE: −0.05610 ± 0.01428; *p* < 0.0001) and significantly increased with increasing relative area of wetlands (WETLA) (0.13550 ± 0.04576; *p* = 0.003) (Figure 4). All considered coefficients were significant (*p* < 0.05).

**Figure 5:**
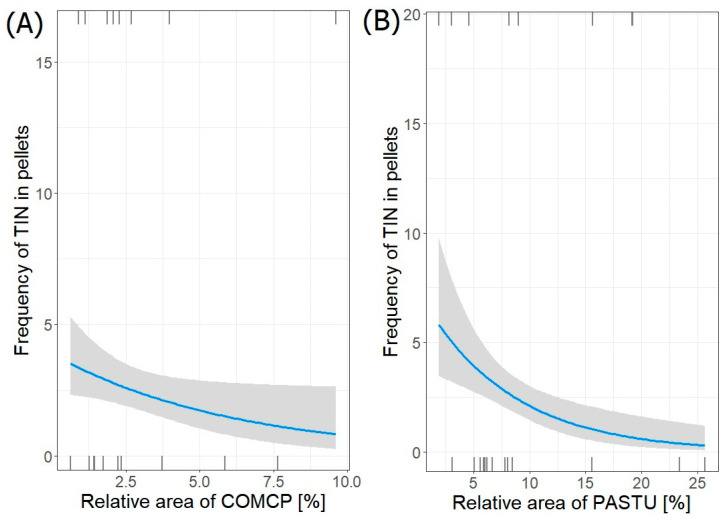
Frequency of terrestrial invertebrate (TIN) in pellets from both regions in relation to relative area of (**A**) COMCP and (**B**) PASTU in foraging areas around heronries. Blue lines indicate GLM fit; rug plots of negative (bottom) and positive (top) residuals.

Only one best-fit model (GLM negative binominal regression) describing the relation between the frequency of occurrence of terrestrial invertebrates (TIN) remains in pellets and its habitats surrounding heronries was selected (Table 8). The frequency of terrestrial invertebrates (TIN) in collected pellets from all colonies (*n =* 51) significantly decreased with both increasing area of complex cultivation patterns (COMCP) (estimate ± SE: −0.16129 ± 0.07952; *p* = 0.043) and relative area of pastures (PASTU) (−0.12592 ± 0.03858; *p* = 0.001) (Figure 5). All considered coefficients were significant (*p* = 0.02).

Only one best-fit model (GLM negative binominal regression) describing the relation between bank vole (MGL) remains in pellets and its habitats surrounding heronries was selected (Table 9). The frequency of bank vole (MGL) in collected pellets from all colonies (*n =* 43) decreased significantly with increasing relative area of non-irrigated arable land (NONIR) (estimate ± SE: −0.02715 ± 0.01109; *p* = 0.01). The coefficient of the model was not significant (*p* > 0.05).

Five best-fit models (GLM negative binominal regression) describing the relation between the frequency of occurrence of short-tailed field vole (MAG) remains in pellets and its habitats surrounding heronries were selected (Table 10). Its frequency in pellets (*n =* 50) in every selected model was not significantly related to any habitat type; only the intercept value was significant (negative binominal regression (estimate ± SE: −1.16199 ± 0.22596); *p* < 0.0001). All coefficients of models with delta < 1 were significant (*p* < 0.05).

**Figure 6:**
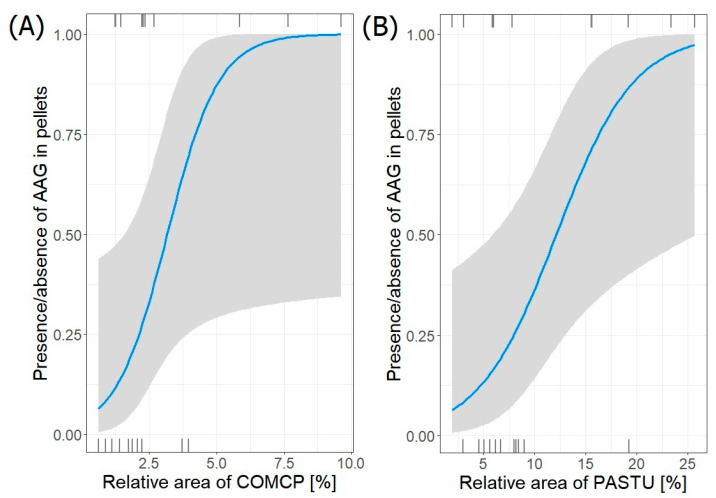
Frequency of striped field mouse (AAG) in pellets from NE region in relation to relative area of (**A**) COMCP and (**B**) PASTU in foraging areas around heronries. Blue lines indicate GLM fit; rug plots of negative (bottom) and positive (top) residuals.

Four best-fit models (binomial model for presence/absence) describing the relation between the frequency of occurrence of striped field mouse (AAG) remains in pellets and its habitats surrounding heronries were selected (Table 11). AAG was present exclusively in colonies in the NE region. The frequency of striped field mouse in pellets (*n =* 43) significantly increased with both increasing relative area of complex cultivation patterns (COMCP) (estimate ± SE: 1.0496 ± 0.5308; *p* = 0.048) and pastures (PASTU) (binomial model for presence/absence; (0.2641 ± 0.1152; *p* = 0.022)) (Figure 6). The coefficients of the first selected model with delta < 1 were significant (*p* < 0.05), while in the rest, they were not significant (*p* > 0.05).

### 3.5. Multivariate Analyses

#### 3.5.1. ANOSIM

ANOSIM indicated significant dissimilarities in diet composition items between the two regions: R value = 0.7821; *p* = 0.0001 (Euclidean distance for permutation N = 9.999).

We did not find any significant differences in habitat area compositions between the two regions: R value = 0.048739; *p* = 0.1717 (Euclidean distance for permutation N = 9.999).

#### 3.5.2. nMDS

**Figure 7:**
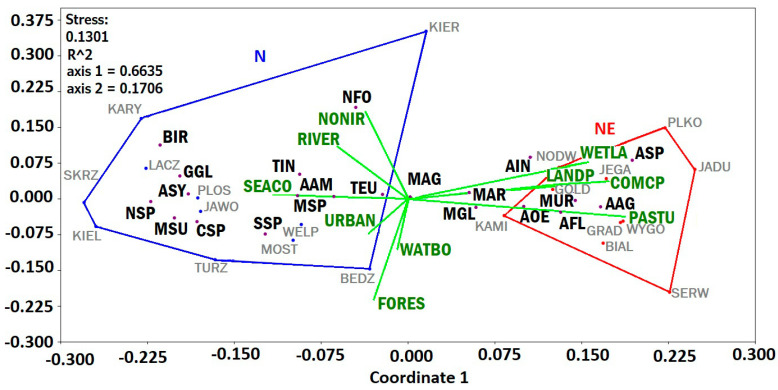
Non-metric multidimensional scaling (NMDS) (with Euclidean dissimilarities matrix) represents encountered prey items from investigated colonies. Each convex hull contains data from one study region. Black 3−letter codes—prey item type; grey 4−letter codes—colony name; green 5−letter codes—habitat type (environmental variables).

The nMDS plot of similarity in the abundance of prey in the pellets of Grey Herons (with an acceptable value of stress: 0.13) showed that samples from N and NE regions clustered in different positions in relation to the first axis (Figure 7).

The first two axes explained 66.4% of the observed variance with a stress value of 13.01% and clustered opposite to the Coordinate 2 axis in the results biplot (explaining 17.1% of observed variance) (Figure 7). A cluster representing the N region included the following prey items: European water vole (AAM), wood mouse (ASY), bird taxa (BIR), species of genus *Crocidura* (CSP), European edible dormouse (GGL), species of genus *Neomys* (NSP), vole sp. (MSP), European pine vole (MSU), Eurasian water shrew (NFO), species of genus *Sorex* (SSP), European mole (TEU), and terrestrial invertebrates (TIN). The cluster representing the NE region includes five prey taxa: striped field mouse (AAG), yellow-necked mouse (AFL), tundra vole (AOE), species of genus *Apodemus* (ASP), and species from the family of Muridae (MUR). Taxa that are outside the convex hulls of both regions are aquatic invertebrates (AIN), short-tailed field vole (MAG), common vole (MAR), and bank vole (MGL).

Coordinate 1 axis variables are as follows: SEACO, NONIR, URBAN, and RIVER, and were associated with the N region, while COMCP, LANDP, PASTU, and WETLA were associated with the NE region. Environmental variables associated with the Coordinate 2 axis, i.e., FORES and WATBO, explained less efficiently observed inter-regional differences in prey item composition (Figure 7). ASP and AIN were strongly related to WETLA areas. AOE, AAG, AFL, MAR, MGL, and MUR are associated with colony surroundings with abundant PASTU areas. MAR was related to areas with abundant COMCP and LANDP. NFO was more common in surroundings with abundant NONIR areas. AAM, MSP, TIN, and TEU were often found in colonies with SEACO areas. The occurrence frequency of MAG remains was similar in every habitat type (Figure 7).

#### 3.5.3. SIMPER

SIMPER indicated five prey items contributing the most to the observed dissimilarities between two regions. AAG and aquatic invertebrates were more common in the NE colonies, whereas European water vole, *Microtus* sp., and terrestrial invertebrates were more common in the N region (Table 12). The average dissimilarity calculated in Euclidean distance was 117.8.

## 4. Discussion

The pellet composition reported in this study is generally similar to other descriptions in the literature [3,6,57,58]. Pellets contained undigested prey parts, mainly comprising mammalian hair; skeletal parts, such as long bones, skulls, or teeth belonging to various species of mammals; and invertebrate remains. Bird feathers were found occasionally, as has been reported in other studies [3,6]. The analysis of the diet composition of a generalist and opportunistic predator like the Grey Heron brings valuable information about habitat conditions and can provide clues about changes occurring within ecosystems impacted by climate change or human pressure on the landscape [59].

Our results suggest opportunistic foraging by Grey Herons on insects and mammals commonly occurring in habitats in the vicinity of colonies. As predicted, the diet of the Grey Heron does exhibit inter-regional differences. The NE region had a significantly higher contribution of areas of farmland habitats (COMCP, LANDP), open grassy areas (PASTU), and wetlands (WETLA) compared to forests (FORES), urbanized areas (URBAN), and non-irrigated arable lands (NONIR) around the N colonies. Noted differences concern areas of optimal foraging grounds, i.e., WETLA, as well as secondary (suboptimal) foraging grounds of the Grey Heron (e.g., open grassy areas, agro-related habitats) [4,14]. However, the contribution of areas of primary (optimal) foraging grounds, such as inland hydrographic features including water bodies (WATBO) and rivers (RIVER), as the only habitat types did not differ between regions. Presumably, the presence of these environments near nesting sites, providing the main food source for adults and young, is important for successful breeding [14,33,34]. In contrast to our expectations, the presence of one region-specific habitat, i.e., SEACO, did not affect the frequency of the prey items we found. However, while comparing coastal and inland colonies in the N region, we noted some differences, with the Eurasian water shrew (NFO) being present exclusively in inland colonies and avian remains (BIR) only in coastal heronries. Coastal areas with high soil salinity, extensive uncovered areas, and poor-nutrient sandy environments constitute a poor medium for the development of most terrestrial species, which are not adapted for living in high-salt surroundings. The coastal zone results, in general, showed lower species biodiversity compared to inland zones characterized by more complex and diverse landscape features. In contrast to results acquired by [5], we found no differences in the frequency of invertebrate remains in collected pellets between inland and coastal heronries. Apparent differences between coastal and inland colonies have been reported for prey items not present in pellets, i.e., fish (e.g., [3]).

Inter-regional differences in available habitat types in close proximity to heronries seem to explain the observed composition dissimilarities of the Grey Heron’s diet, especially as this species is considered an opportunistic feeder [4,6,14,60]. Pellets collected in the NE region had fewer prey types (*n =* 13), three of which were present only within this region, compared to the N region (*n =* 18), with eight exclusive prey taxa. Aquatic invertebrates (AIN), yellow-necked mouse (AFL), tundra vole (AOE), and striped field mouse (AAG) appeared more often in pellets from the NE region, where striped field mouse (AAG) occurred abundantly and exclusively within this region. It has been found that AIN are related to wetlands areas [61], being more abundant in this region. AAG, similar to AFL and AOE, is associated with grassy areas, pastures, wetlands, woodland shrubs, and forest habitats [62,63,64], which are also commonly found in close proximity to colonies from the NE region. European water vole (AAM), *Microtus* sp. (MSP), and terrestrial invertebrates (TIN) were more common in pellets from the N region.

Considering the local spatial scale of habitats around particular colonies, we found significant relationships between some prey items and four types of habitats: two agro-related habitats (COMCP, NONIR); grassy, open-space environments (PASTU); forested areas (FORES); and wetlands (WETLA). The frequencies of European water vole (AAM) and terrestrial invertebrates (TIN) decreased, while striped field mouse (AAG) increased with increasing areas of COMCP and PASTU habitats. Presumably, the lower frequency of AAM and TIN in COMCP and PASTU results from their avoidance of open environments, caused by increased exposure to predators because of no vegetation cover [65]. The striped field mouse (AAG), on the other hand, was more common in open environments due to the species’ environmental preferences [63]. *Microtus* sp. (MSP) was more often noted in pellet material collected from areas with more extensive NONIR and FORES habitats. The frequency of the bank vole (MGL) remains in pellets, in contrast to TIN, decreased with the growing contribution of the NONIR habitat in proximity to heronries. This can be explained by the habitat preferences of local MGL populations towards more humid environments, such as wetlands or inland bodies of water [65]. The remains of aquatic invertebrates (AIN) in pellets were less frequent in areas with extensive forests (FORES), while their abundance grew with increasing area of wetlands (WETLA), possibly due to their strict relation to aquatic-related environments [61].

We are aware of some limitations of our study. First, we only considered non-fish components of the Grey Heron diet, which makes up only a minor part of the biomass contribution in European populations (e.g., [3,15]). Second, we considered two regions with generally similar postglacial landscapes. Besides the similar geomorphological history of the two study sites, one habitat (SEACO) is present exclusively in the northern region. Also, the Vistula River has constituted a post-glacial era geographic barrier for gene flow and the general distribution of small mammals [66], which could also be reflected in inter-regional diet composition differences. Third, in the case of some taxa, we were not able to identify some remains to the species level. Thus, we were forced to pull some remains into higher taxonomic categories, which may have blurred some patterns of potential differences. Finally, we did not consider intra- and inter-specific competition for prey. The influence of these two agonistic interactions could be reflected in shifts in the non-fish dietary components of the Grey Heron. Undoubtedly, more research and the consideration of other factors such as population size, species composition, or distance to urban areas are needed to better understand what factors impact the heron diet. Nevertheless, we believe that our study still provides valuable insight not only into the diet composition of the Grey Heron but also insight into the relationships between prey items and available habitats in the vicinity of heronries.

## 5. Conclusions

In conclusion, we found some differences exist in the diet of Grey Herons between two regions of Poland (N, NE), expressed by the presence/absence of some prey item types. We also found some differences in diet composition at the local scale, i.e., between particular colonies and the composition of foraging habitats (farmlands, pastures, forests, and wetlands) around them. The proportions of inland hydrographic features, i.e., bodies of water and rivers around colonies, were similar in both regions, indicating their importance for the Grey Heron and functioning as optimal foraging grounds. Still, more research is needed to fully understand the topic of spatial variation in the diet of Grey Herons.

## Figures and Tables

**Table 1:** List of distinguished prey item categories. “-” stands for unused naming method for prey item. List of prey items is sorted alphabetically by three-letter code.

Species	Scientific Name	Code
striped field mouse	*Apodemus agrarius*	AAG
European water vole	*Arvicola amphibius*	AAM
yellow-necked mouse	*Apodemus flavicollis*	AFL
aquatic invertebrates	-	AIN
tundra vole	*Alexandromys oeconomus*	AOE
species of genus *Apodemus*	*Apodemus* sp.	ASP
wood mouse	*Apodemus sylvaticus*	ASY
bird taxa	-	BIR
species of genus *Crocidura*	*Crocidura* sp.	CSP
European edible dormouse	*Glis glis*	GGL
short-tailed field vole	*Microtus agrestis*	MAG
common vole	*Microtus arvalis*	MAR
bank vole	*Myodes glareolus*	MGL
*voles*	*Microtus* sp.	MSP
European pine vole	*Microtus subterraneus*	MSU
species from family of Muridae	Muridae	MUR
Eurasian water shrew	*Neomys fodiens*	NFO
species of genus *Neomys*	*Neomys* sp.	NSP
species of genus *Sorex*	*Sorex* sp.	SSP
European mole	*Talpa europaea*	TEU
terrestrial invertebrates	-	TIN

**Table 2:** Codes for hydrographic and inland habitat features with short characteristics [31].

Code (Habitat)	Short Characteristics
COMCP (Complex cultivation patterns)	Juxtaposition of small parcels of annual crops, city gardens, pastures, fallow lands, and/or permanent crops somewhere with scattered houses.
FORES (Forests)	Broad-leaved, coniferous, and mixed forests combined.
LANDP (Land principally occupied by agriculture, with significant areas of natural vegetation)	Land occupied by irrigated cultivated parcels interspersed with significant areas of natural or semi-natural vegetation, e.g., forests, shrubs, vineyards, orchards, or plantations.
NONIR (Non-irrigated arable land)	Land occupied mainly by rainfed cultivated parcels with a crop rotation system.
PASTU (Pastures)	Dense grass cover of floral composition, dominated by Poaceae, not under a rotation system. Areas under intense human disturbance are included, mainly for grazing, but the folder may be harvested mechanically. Areas sparsely covered in vegetation or transitional woodland-shrub included.
RIVER (Rivers)	Natural or artificial water courses serve as water drainage channels. Includes canals and rivers that have been canalized.
SEACO (Seacoast)	Area of coastal zone of the Baltic Sea (2 m wide).
URBAN (Urban zones)	Land covered by structures and the transport network (roads, motorways, and railways, including associated installations, e.g., stations), buildings, artificially surfaced areas (e.g., asphalt), infrastructure of port areas, marinas, airports, dump sites, mineral extraction sites, green urban areas, and industrial fabric structures.
WATBO (Water bodies)	Natural or artificial stretches of water, also with low floating aquatic vegetation, archipelago of lakes inside land areas, coastal lagoons, and fish farms.
WETLA (Inland wetlands)	Low-lying land (inland wetlands, marshes, swamps) usually flooded in winter and more or less saturated by water all year round. Covered by a specific low ligneous, semi-ligneous, or herbaceous vegetation.

**Table 3:** Significant regional differences in prey item composition.

Prey Item	Regional Prevalence	Counts for Regions		Difference N vs. NE	
		N	NE	Test Value	*p*
European water vole (AAM)	N	97	43	10.086	0.001
*Microtus* sp. (MSP)	N	56	21	9.899	0.002
aquatic invertebrates (AIN)	NE	18	45	11.372	0.001
terrestrial invertebrates (TIN)	N	39	12	10.264	0.001
tundra vole (AOE)	NE	4	15	-	0.015
yellow-necked mouse (AFL)	NE	2	9	-	0.03

**Table 4:** Relative proportions of habitats around Grey Heron breeding colonies in two regions. Difference—G test scores (calculations were performed on raw data in km^2^). Significant differences are bolded.

Habitat	Area [%]		Difference	
	N	NE	G	*p*
COMCP	**1.6**	**3.7**	**102.75**	**<0.0001**
FORES	**34.0**	**29.9**	**25.05**	**<0.0001**
LANDP	**4.8**	**10.6**	**298.96**	**<0.0001**
NONIR	**44.0**	**35.2**	**105.05**	**<0.0001**
PASTU	**7.8**	**12.4**	**145.2**	**<0.0001**
RIVER	0.4	0.3	3.1985	0.07
SEACO	0.05	0	Not analyzed	-
URBAN	**4.4**	**2.25**	**19.717**	**<0.0001**
WATBO	2.5	2.2	1.1409	0.2855
WETLA	**0.5**	**2.48**	**187.86**	**<0.0001**

**Table 5:** A list of all best-fit GLM models (ΔAICc < 1) investigating relations between frequency of occurrence of water vole (AAM) and its surrounding habitats. Model with the lowest ΔAICc is bolded.

**Model**	**df**	**logLik**	**AICc**	**ΔAICc**	**Weight**
AAM~COMCP + PASTU	**3**	**−43.370**	**94.2**	**0.00**	**0.618**
AAM~COMCP + PASTU + SEACO	4	−42.305	95.1	0.96	0.382

**Table 6:** Best-fit GLM model (ΔAICc < 1) investigating relations between frequency of occurrence of vole sp. (MSP) and its surrounding habitats. Model with the lowest ΔAICc is bolded.

Model	df	logLik	AICc	Delta	Weight
MSP~FORES + NONIR	**3**	**−42.747**	**92.9**	**0.00**	**1.000**

**Table 7:** Best-fit GLM model (ΔAICc < 1) investigating relations between frequency of occurrence of aquatic invertebrates (AIN) and their surrounding habitats. Model with the lowest ΔAICc is bolded.

Model	df	logLik	AICc	Delta	Weight
AIN~FORES + WETLA	**3**	**−40.792**	**89**	**0.00**	**1.000**

**Table 8:** Best-fit GLM model (ΔAICc < 1) investigating relations between frequency of occurrence of terrestrial invertebrates (TIN) and their surrounding habitats. Model with the lowest ΔAICc is bolded.

Model	df	logLik	AICc	Delta	Weight
TIN~COMCP + PASTU	**3**	**−35.889**	**79.2**	**0.00**	**1.000**

**Table 9:** Best-fit GLM model (ΔAICc < 1) investigating relations between frequency of occurrence of bank vole (MGL) and its surrounding habitats. Model with the lowest ΔAICc is bolded.

Model	df	logLik	AICc	Delta	Weight
MGL~NONIR	**2**	**−31.987**	**68.6**	**0.00**	**1.000**

**Table 10:** A list of all best-fit GLM models (ΔAICc < 1) investigating relations between frequency of occurrence of short-tailed field vole (MAG) and its surrounding habitats. Model with the lowest ΔAICc is bolded.

Model	df	logLik	AICc	Delta	Weight
MAG~URBAN	**2**	**−32.108**	**68.9**	**0.00**	**0.237**
MAG~INT	1	−33.371	69.0	0.07	0.229
MAG~SEACO	2	−32.219	69.1	0.22	0.212
MAG~LANDP	2	−32.412	69.5	0.61	0.175
MAG~PASTU + SEACO	3	−31.206	69.8	0.94	0.148

**Table 11:** A list of all best-fit GLM models (ΔAICc < 1) investigating relations between frequency of occurrence of striped field mouse (AAG) and its surrounding habitats. Model with the lowest ΔAICc is bolded.

Model	df	logLik	AICc	Delta	Weight
AAG~COMCP + PASTU	**3**	**−8.387**	**24.2**	**0.00**	**0.321**
AAG~LANDP + PASTU	3	−8.606	24.6	0.44	0.258
AAG~COMCP + SEACO	3	−8.743	24.9	0.71	0.225
AAG~SEACO + WETLA	3	−8.881	25.2	0.99	0.196

**Table 12:** SIMPER results of the highest contribution to group differences (above 10%) for five prey items.

Taxon (Code)	Av. Dissim.	Contrib. %	Cumulative %	Mean N	Mean NE
water vole (AAM)	24.79	21.05	21.05	8.82	4.3
aquatic invertebrates (AIN)	21.01	17.84	38.89	1.64	4.5
striped field mouse (AAG)	19.5	16.56	55.45	0	4.3
*Microtus* sp. (MSP)	16.55	14.06	69.5	5.09	2.1
terrestrial invertebrates (TIN)	11.78	10	79.51	3.55	1.2

## Data Availability

Data are contained within the article and Supplementary Materials.

## Supplementary Materials

The following supporting information can be downloaded at: https://www.mdpi.com/article/10.3390/ani14172461/s1, S1—list of analyzed colonies, name abbreviations, and their localization.
